# Bicycling for Women

**Published:** 1895-08

**Authors:** 


### Editorial.

### BICYCLING FOR WOMEN.

A local preacher who enjoys some notoriety as a sensationalist and a *poseur* has taken it upon himself, with all the callow rhetoric of a country schoolmaster, to denounce the bicycle as a most diabolical invention, especially designed and executed for the subversion of the morals of women. In the face of such anathemas hurled from the very horns of the altar, she must be a brave woman indeed who would continue in the error of her way.

If the fervid eloquence of the very reverend doctor had been tempered with cold facts, the way of the gentle transgressor would not have seemed so hard. These facts, so conspicuously absent in the clerical denunciation of the bicycle, are well set forth in our ever dignified and reliable contemporary, the Chicago *Medical Standard*, and apply both to male and female offenders. In a recent editorial on this subject it says: "A very severe indictment can be brought against bicycle abuse, but these abuses are common to a hundred methods of outdoor exercise. There is no necessary emotional excitement about cycling. Despite its widespread use, racing is rarer with the wheel than with the horse. 'Scorching' has been largely, if not entirely, responsible for all evils charged to the wheel. Wheel kyphosis is a result of bad position and, as women cyclers have shown, avoidable. A very calm, judicial critic, Dr. Justus Championiere, declares that for grace and regularity of motion woman is man's superior in the use of the bicycle. She cycles as well as she dances.

"To the sedentary clerk the wheel has brought fresh air and exercise previously impossible from the cost of horses. By hygienic dress the wheel has benefitted woman to an extent which far outweighs any possible evils resultant on its use. It has doomed the corset and waist-attached skirts, the source of unlimited pelvic evils. The ease and freedom with which outdoor exercise is obtained has tempted into the sunshine women with moping tendencies to invalidism. To none is it likely to prove a greater boon than to the farmer woman. Among these, because of isolation, nervous disease and insanity are rife. Decades ago Dr. Patterson, who had studied insanity in Iowa, Ohio, Indiana and Illinois farming regions, remarked: 'The wives and daughters of farmers during inclement seasons have fewer comforts connected with outdoor life and less adequate protection from cold and humid air than the women who live in our towns and cities.' It is probable, taking prairie farm life with all its surroundings, that the average standard of vital force in those who live upon farms is below that of those who live in cities and towns. It must not be inferred, however, from these suggestions that the noble and pleasing pursuits of agriculture favor the production of insanity. The errors of living and the discomforts alluded to are not necessarily connected with, and certainly not limited to, farm life. Precedent to insanity always occur malaise and auto-intoxication, with resultant hepatic, renal and gastro-intestinal disorders and consequent cardiac stress. Much of the so-called dyspepsia of farmers is gastro-neurasthenia. Open-air exercise (not athletics) and visiting, which the bicycle can secure, break up the dead routine underlying invalidism. Bicycling, like all exercise, has its dangers, but it has become of such practical use that there are less proportionately than those of other modes. To point out abuses, is to prevent them."

The most important objection to bloomers is their unsightliness. No woman can afford to make herself ridiculous, and with the use of bloomers, they come perilously near it. There can be no doubt but that this garb is merely transitional, and is designed as a compromise measure before the introduction of the more sensible knickerbockers.

*Mens sana in corpore sano.* Let the women ride.

---

We have received the Seventeenth Annual Announcement of the Southern Medical College, of Atlanta, for the session of 1895–96. The course of instruction in this institution has been broadened from year to year, instructors and assistants added and the facilities for teaching have been advanced to a very high point, and the Southern Medical College has now been placed in the foremost rank of the medical schools of the South. We wish it a continuation of the prosperity and success which it has enjoyed and richly merited in the past.

---

DR. J. ALLISON HODGES, Professor of Anatomy and of Diseases of the Brain and Nervous System in the University College of Medicine, Richmond, Va., is to be congratulated on the good reports received of his address during the recent commencement exercises of Davidson College, N. C. He remains of course, Corresponding Secretary of the University College of Medicine, Richmond, Va.

---

The Vanderbilt Medical College, of Nashville, Tenn., is just completing an elegant new building, an excellent cut of which is represented on another page.

The faculty contains some of the best known medical men in the South. Among others are Richard Douglas, so well and favorably known to the profession for his excellent work in diseases of women and abdominal surgery; Duncan Eve, whose family name has been so closely connected with medical progress in the South for eighty years; G. C. Savage, the talented editor of the *Ophthalmic Record*; W. M. Late Coplin, who has recently accepted the chair of pathology and bacteriology.

Under such teachers, the course of instruction cannot be otherwise than of a very high grade.

---

MEDICAL DEPARTMENT OF UNIVERSITY OF NASHVILLE was inaugurated 1851 with a faculty of eminent men, and its alumni have furnished the profession with men of high rank in war as in peace. For some years since the war it was operated in connection with Vanderbilt University; but hereafter it will again resume its independent position. The old building has been torn down and a new one—commodious and complete in every detail—is about completed, just opposite the University of Nashville campus.. Its reorganized faculty consists largely of the immediate successors of the founders of the school, besides a few new professors of noted ability as medical teachers.

